# Mendelian sampling covariability of marker effects and genetic values

**DOI:** 10.1186/s12711-016-0214-0

**Published:** 2016-04-23

**Authors:** Sarah Bonk, Manuela Reichelt, Friedrich Teuscher, Dierck Segelke, Norbert Reinsch

**Affiliations:** Institute of Genetics and Biometry, Leibniz-Institute for Farm Animal Biology (FBN), Wilhelm-Stahl-Allee 2, 18196 Dummerstorf, Germany; Vereinigte Informationssysteme Tierhaltung w.V., Heideweg 1, 27283 Verden, Germany

## Abstract

**Background:**

Measures of the expected genetic variability among full-sibs are of practical relevance, such as in the context of mating decisions. An important application field in animal and plant breeding is the selection and allocation of mates when large or small amounts of genetic variability among offspring are desired, depending on user-specific goals. Estimates of the Mendelian sampling variance can be obtained by simulating gametes from parents with known diplotypes. Knowledge of recombination rates and additive marker effects is also required. In this study, we aimed at developing an exact method that can account for both additive and dominance effects.

**Results:**

We derived parent-specific covariance matrices that exactly quantify the within-family (co-)variability of additive and dominance marker effects. These matrices incorporate prior knowledge of the parental diplotypes and recombination rates. When combined with additive marker effects, they allow the exact derivation of the Mendelian sampling (co-)variances of (estimated) breeding values for several traits, as well for the aggregate genotype. A comparative analysis demonstrated good average agreement between the exact values and the simulation results for a practical dataset (74,353 German Holstein cattle).

**Conclusions:**

The newly derived method is suitable for calculating the exact amount of intra-family variation of the estimated breeding values and genetic values (comprising additive and dominance effects).

## Background

The degree of genetic variability among full-sibs is known as Mendelian sampling variance. This variability is due to the inheritance of random samples of alleles from both parents. For a quantitative trait, the amount of this variability depends on the parental degree of heterozygosity, $1-F_{{\mars}}$, where $F_{{\mars}}$ ($F_{{\venus}}$) is the inbreeding coefficient of an individual’s sire (dam), which is derived from the pedigree. Under additivity and with unlinked loci, the Mendelian sampling variance is the sum of two parental contributions, $\frac{1}{4}\sigma ^2_a (1-F_{{\mars}})+ \frac{1}{4}\sigma ^2_a (1-F_{{\venus}})$, where $\sigma ^2_a$ is the additive genetic variance [1]. The latter expression is of general importance in quantitative genetics, especially in the context of estimating genetic parameters and in genetic evaluations. In certain models (e.g. [2, 3]), it is used explicitly for the relative weighting of observations from progeny of inbred versus non-inbred parents. Moreover, the inverse Mendelian sampling variance plays a pivotal role in direct inversion of the numerator relationship matrix [4].

New methods to track Mendelian sampling variance are based on the availability of phased genotypes (diplotypes) at genetic markers across the genome as a byproduct of genomic selection (e.g. [5, 6]). Single nucleotide polymorphism (SNP) diplotypes of parents differ in terms of three features: the degree of heterozygosity, the genotypes at homozygous loci, and the linkage phase between loci. All of these features have consequences for the variability of gametes that are generated by a particular individual, and thus for the variance among the progeny in a family. A small within-family genetic variation contributes to phenotypic uniformity, which is desired e.g. for birth-weight of piglets (e.g. [7]), while a large Mendelian sampling variability may increase selection opportunity between sibs [6]. When phased genotypes are available, it is possible to simulate a large sample of the population of gametes of a selection candidate by considering recombination within chromosomes, as was demonstrated in a study on 58,035 Holsteins [5]. However, to the best of our knowledge, exact formulae for calculating the Mendelian sampling variance from phased genotypes have not been reported previously.

In this study, we provide the requisite formulae for the exact calculation of within-family genetic variation. The within-family covariance matrix between the additive and dominance effects of all markers can be derived exactly from phased SNP-genotypes and the known genetic distances between markers. Conversion to the within-family variance of (estimated) additive and dominance values for a trait is then achieved via the (estimated) additive and dominance marker effects. We provide a comparison between the results obtained by simulations and the exact method, as well as a brief discussion of the application of this method to the allocation of mates.

## Methods

In the following, a breeding population is assumed, where phased SNP-genotypes are available for all potential mating partners, as well as estimates of the SNP-effects for all traits. Furthermore, it is assumed that the genetic distances between all markers are known in terms of their recombination rates, which are summarized in a comprehensive genetic map for all SNPs.

In the first part of this section, we only consider additive marker effects that contribute to the genomic breeding value of an individual:

1 $$g ={{\mathbf{c}}^{\prime}} {\mathbf{m}},$$

where ${\mathbf{c}}^{\prime }$ is a row vector of genotype indicators (see Eq. 3) and ${\mathbf{m}}$ is a vector of marker effects. We show that the within-family covariance matrix ${\varvec{\Omega}}$ of ${\mathbf{c}}$, with dimensions equal to the number of marker effects, can be expressed as the sum of two parent-specific covariance matrices ${\varvec{\Omega}}^{{\mars}}$ and ${\varvec{\Omega}}^{{\venus}}$. They define independent parental contributions to the Mendelian sampling variance of genomic breeding values of a trait:

2 $$\sigma ^2_g = {\mathbf{m}}^{\prime } {\varvec{\Omega }} {\mathbf{m}}.$$

Subsequently we demonstrate that this additive property of the covariance matrix vanishes when dominance marker effects are included.

### Definitions and the case of pure additivity

Throughout this study, we exploit the fact that correlations between marker effects are equivalent to correlations between genotype indicators. For the additive effect *a* (half the average phenotypic difference between homozygotes) at a two-allelic (*A*, *B*) locus *i*, the genotype indicator $c_{a,i}$ is given by:

3 $$\begin{aligned} c_{a,i}=\left\{ \begin{array}{ll} 1, &{}\quad \text{ for}\,\text{ genotype } AA \\ 0, &{}\quad \text{ for}\,\text{ genotypes}\,AB \text{ or } BA \\ -1, &{}\quad \text{ for}\, \text{ genotype }\, BB \end{array}\right.{,} \end{aligned}$$

and for dominance effects by:

4 $$\begin{aligned} c_{d,i}=\left\{ \begin{array}{ll} -1, &{}\quad \text{ for }\, \text{ genotypes }\, AA\, \text{ or }\,BB\\ 1, &{}\quad \text{ for } \,\text{ genotypes }\, AB\,\text{ or }\,BA \end{array}\right.{,} \end{aligned}$$

for $1\le i \le n$, where *n* is the number of markers. See the discussion for a treatment of the coding of additive and dominance effects. Derivation of the covariance is based on the linkage disequilibrium (LD) among all the gametes produced by a parent with a particular diplotype.

A parent has one of the 16 possible diplotypes when two bi-allelic markers are involved, which are represented in Table 1. Pairs of additive and dominance genotype indicators are given in columns 2 to 5. The gametes generated by the parent (comprising one allele at the first locus and one allele at the second locus) follow a probability distribution that depends on the diplotype of the parent. Columns 6 to 9 shows the probabilities of gametes, to which we apply the concept of LD. For each parent, LD can be determined as:

5 $$D_{i,j}= p_{A-A} p_{B-B}- p_{A-B} p_{B-A},$$

where the lower index indicates gametes (see Table 1, column 10).

**Table 1:** Ten classes of parental diplotypes with different two-locus genotypes and distributions of produced gametes

Parental diplotype	Genotype indicators				Probabilities of gametes				Characterizing parameters		
	$c_{a,i}$	$c_{a,j}$	$c_{d,i}$	$c_{d,j}$	$p_{A-A}$	$p_{A-B}$	$p_{B-A}$	$p_{B-B}$	$D_{i,j}$	$p_{i}$	$p_{j}$
$$\begin{array}{c} A-A \\ A-A \end{array}$$	1	1	−1	−1	1	0	0	0	0	1	1
$$\begin{array}{c} A-A \\ A-B \end{array}/\begin{array}{c} A-B \\ A-A \end{array}$$	1	0	−1	1	$\frac{1}{2}$	$\frac{1}{2}$	0	0	0	1	$\frac{1}{2}$
$$\begin{array}{c} A-A \\ B-A \end{array}/\begin{array}{c} B-A \\ A-A \end{array}$$	0	1	1	−1	$\frac{1}{2}$	0	$\frac{1}{2}$	0	0	$\frac{1}{2}$	1
$$\begin{array}{c} A-B \\ A-B \end{array}$$	1	−1	−1	−1	0	1	0	0	0	1	0
$$\begin{array}{c} B-A \\ B-A \end{array}$$	−1	1	−1	−1	0	0	1	0	0	0	1
$$\begin{array}{c} A-A \\ B-B \end{array}/\begin{array}{c} B-B \\ A-A \end{array}$$	0	0	1	1	$\frac{1-\theta _{i,j}}{2}$	$\frac{\theta _{i,j}}{2}$	$\frac{\theta _{i,j}}{2}$	$\frac{1-\theta _{i,j}}{2}$	$\frac{1-2\theta _{i,j}}{4}$	$\frac{1}{2}$	$\frac{1}{2}$
$$\begin{array}{c} A-B \\ B-A \end{array}/\begin{array}{c} B-A \\ A-B \end{array}$$	0	0	1	1	$\frac{\theta _{i,j}}{2}$	$\frac{1-\theta _{i,j}}{2}$	$\frac{1-\theta _{i,j}}{2}$	$\frac{\theta _{i,j}}{2}$	$-\frac{1-2\theta _{i,j}}{4}$	$\frac{1}{2}$	$\frac{1}{2}$
$$\begin{array}{c} A-B \\ B-B \end{array}/\begin{array}{c} B-B \\ A-B \end{array}$$	0	−1	1	−1	0	$\frac{1}{2}$	0	$\frac{1}{2}$	0	$\frac{1}{2}$	0
$$\begin{array}{c} B-A \\ B-B \end{array}/\begin{array}{c} B-B \\ B-A \end{array}$$	−1	0	−1	1	0	0	$\frac{1}{2}$	$\frac{1}{2}$	0	0	$\frac{1}{2}$
$$\begin{array}{c} B-B \\ B-B \end{array}$$	−1	−1	−1	−1	0	0	0	1	0	0	0

Different diplotypes with identical genotypes at markers *i* and *j* are separated by a slash. Genotype indicators are given for additive ($c_{a,i},c_{a,j}$) and dominance ($c_{d,i},c_{d,j}$) effects at both parental marker genotypes. The probabilities of gametes are specific for each diplotype class and they can be summarized by three characteristic parameters: LD $D_{i,j}$ and the probabilities $p_{i}$, $p_{j}$ for an *A*-allele at locus *i* or *j*. $\theta _{i,j}$ is the recombination fraction

In Table 1 the probability of the appearance of allele *A* at the *i*th locus is denoted by $p_i$. Note that this definition applies to the diplotypes as well as to the gametes of the parent. The values for $p_i$ and $p_j$ are given in columns 11 and 12 of Table 1. All entries in Table 1 apply to both the sire (upper index ${\mars}$) and the dam (upper index ${\venus}$).

The joint distribution of genotypes at two bi-allelic marker loci among offspring is outlined in Table 2, using the frequencies of the parental gametes from Table 1. All nine two-locus genotypes are enumerated, together with their underlying diplotypes (the upper haplotype is paternal; columns 1–3). The probability of each ordered diplotype is the product of the two gametic probabilities, and the probability of a two-locus genotype is the sum of the probabilities for all of its possible underlying diplotypes (Table 2, last column). In the next step, the sex-specific probabilities (indexed as $\mars$ or $\venus$) of the parental gametes are expressed as functions of the parent-specific LD-parameters and allele frequencies:

6 $$p^{{\mars}}_{A-A}= {} D^{{\mars}}_{i,j}+p^{{\mars}}_i p^{{\mars}}_j,$$

7 $$p^{{\mars}}_{A-B}= {} -D^{{\mars}}_{i,j}+p^{{\mars}}_i \left( 1-p^{{\mars}}_j\right) ,$$

8 $$p^{{\mars}}_{B-A}= {} -D^{{\mars}}_{i,j}+\left( 1-p^{{\mars}}_i\right) p^{{\mars}}_j,$$

and

9 $$p^{{\mars}}_{B-B}=D^{{\mars}}_{i,j}+\left( 1-p^{{\mars}}_i)(1- p^{{\mars}}_j\right) ,$$

for a sire. Expressions for a dam are obtained in the same manner by replacing ${\mars}$ with ${\venus}.$

**Table 2:** Two-locus genotype probabilities in a full-sib family

L1	L2	Diplotypes	Probabilities
*BB*	*BB*	$$\begin{array}{c} B-B \\ B-B \end{array}$$	$p^{{\mars}}_{B-B}p^{{\venus}}_{B-B}$
	*AB*/*BA*	$$\begin{array}{c} B-A \\ B-B \end{array}/\begin{array}{c} B-B \\ B-A \end{array}$$	$p^{{\mars}}_{B-A}p^{{\venus}}_{B-B}+p^{{\mars}}_{B-B}p^{{\venus}}_{B-A}$
	*AA*	$$\begin{array}{c} B-A \\ B-A \end{array}$$	$p^{{\mars}}_{B-A}p^{{\venus}}_{B-A}$
*AB*/*BA*	*BB*	$$\begin{array}{c} A-B \\ B-B \end{array}/\begin{array}{c} B-B \\ A-B \end{array}$$	$p^{{\mars}}_{A-B}p^{{\venus}}_{B-B}+p^{{\mars}}_{B-B}p^{{\venus}}_{A-B}$
	*AB*/*BA*	$$\begin{array}{c} A-A \\ B-B \end{array}/\, \begin{array}{c} B-B \\ A-A \end{array}/\, \begin{array}{c} A-B \\ \, B-A \end{array}/\,\begin{array}{c} B-A \\ \, A-B \end{array}$$	$p^{{\mars}}_{A-A}p^{{\venus}}_{B-B}+p^{{\mars}}_{B-B}p^{{\venus}}_{A-A}+p^{{\mars}}_{A-B}p^{{\venus}}_{B-A}+p^{{\mars}}_{B-A}p^{{\venus}}_{A-B}$
	*AA*	$$\begin{array}{c} A-A \\ B-A \end{array}/\begin{array}{c} B-A \\ A-A \end{array}$$	$p^{{\mars}}_{A-A}p^{{\venus}}_{B-A}+p^{{\mars}}_{B-A}p^{{\venus}}_{A-A}$
*AA*	*BB*	$$\begin{array}{c} A-B \\ A-B \end{array}$$	$p^{{\mars}}_{A-B}p^{{\venus}}_{A-B}$
	*AB*/*BA*	$$\begin{array}{c} A-A \\ A-B \end{array}/\begin{array}{c} A-B \\ A-A \end{array}$$	$p^{{\mars}}_{A-A}p^{{\venus}}_{A-B}+p^{{\mars}}_{A-B}p^{{\venus}}_{A-A}$
	*AA*	$$\begin{array}{c} A-A \\ A-A \end{array}$$	$p^{{\mars}}_{A-A}p^{{\venus}}_{A-A}$

Nine classes of two-locus genotypes (L1, L2) in the offspring, which all correspond to ordered diplotypes (separated by a slash, where the upper haplotype is paternal) and the probability of each class as a function of the frequencies of parental gametes (superscripts indicate the sex of the parent and subscripts indicate the haplotypes of gametes)

These terms also allow us to rewrite the genotype probabilities of Table 2 as functions of the LD-parameters and allele frequencies (see Table 3). We consider that they are arranged in a three-by-three matrix ${\mathbf {Z}}=(z_{s,q})$, the rows and columns of which pertain to the genotypes at the first locus and the genotypes at the second locus, respectively (the order of genotypes is *BB*, *AB* / *BA*, and *AA* for both loci); for example, the probability of *BB*, *BB* is:

10 $$\begin{aligned} z_{1,1}&=p^{{\mars}}_{B-B}p^{{\venus}}_{B-B} \nonumber \\&=\left[ D^{{\mars}}_{i,j}+\left( 1-p^{{\mars}}_i\right) \left( 1- p^{{\mars}}_j\right) \right] \nonumber \\&\quad \cdot \left[ D^{{\venus}}_{i,j}+\left( 1-p^{{\venus}}_i\right) \left( 1- p^{{\venus}}_j\right) \right] . \end{aligned}$$

**Table 3:** Two-locus genotype probabilities as functions of characteristic parameters

L1	L2	Probabilities
*BB*	*BB*	$[D^{{\mars}}_{i,j}+(1-p^{{\mars}}_i)(1- p^{{\mars}}_j)][D^{{\venus}}_{i,j}+(1-p^{{\venus}}_i)(1- p^{{\venus}}_j)]$
	*AB*/*BA*	$[-D^{{\mars}}_{i,j}+(1-p^{{\mars}}_i) p^{{\mars}}_j] [D^{{\venus}}_{i,j}+(1-p^{{\venus}}_i)(1- p^{{\venus}}_j)]+[D^{{\mars}}_{i,j}+(1-p^{{\mars}}_i)(1- p^{{\mars}}_j)][-D^{{\venus}}_{i,j}+(1-p^{{\venus}}_i) p^{{\venus}}_j]$
	*AA*	$[-D^{{\mars}}_{i,j}+(1-p^{{\mars}}_i) p^{{\mars}}_j][-D^{{\venus}}_{i,j}+(1-p^{{\venus}}_i) p^{{\venus}}_j]$
*AB*/*BA*	*BB*	$[-D^{{\mars}}_{i,j}+p^{{\mars}}_i (1-p^{{\mars}}_j)][D^{{\venus}}_{i,j}+(1-p^{{\venus}}_i)(1- p^{{\venus}}_j)]+[D^{{\mars}}_{i,j}+(1-p^{{\mars}}_i)(1- p^{{\mars}}_j)][-D^{{\venus}}_{i,j}+p^{{\venus}}_i (1-p^{{\venus}}_j)]$
	*AB*/*BA*	$$\begin{aligned}&[D^{{\mars}}_{i,j}+p^{{\mars}}_i p^{{\mars}}_j][D^{{\venus}}_{i,j}+(1-p^{{\venus}}_i)(1- p^{{\venus}}_j)]+[D^{{\mars}}_{i,j}+(1-p^{{\mars}}_i)(1- p^{{\mars}}_j)][D^{{\venus}}_{i,j}+p^{{\venus}}_i p^{{\venus}}_j]\\&\quad+[-D^{{\mars}}_{i,j}+p^{{\mars}}_i (1-p^{{\mars}}_j)][-D^{{\venus}}_{i,j}+(1-p^{{\venus}}_i) p^{{\venus}}_j]+[-D^{{\mars}}_{i,j}+(1-p^{{\mars}}_i) p^{{\mars}}_j][-D^{{\venus}}_{i,j}+p^{{\venus}}_i (1-p^{{\venus}}_j)]\end{aligned}$$
	*AA*	$[D^{{\mars}}_{i,j}+p^{{\mars}}_i p^{{\mars}}_j][-D^{{\venus}}_{i,j}+(1-p^{{\venus}}_i) p^{{\venus}}_j]+[-D^{{\mars}}_{i,j}+(1-p^{{\mars}}_i) p^{{\mars}}_j][D^{{\venus}}_{i,j}+p^{{\venus}}_i p^{{\venus}}_j]$
*AA*	*BB*	$[-D^{{\mars}}_{i,j}+p^{{\mars}}_i (1-p^{{\mars}}_j)][-D^{{\venus}}_{i,j}+p^{{\venus}}_i (1-p^{{\venus}}_j)]$
	*AB*/*BA*	$[D^{{\mars}}_{i,j}+p^{{\mars}}_i p^{{\mars}}_j][-D^{{\venus}}_{i,j}+p^{{\venus}}_i (1-p^{{\venus}}_j)]+[-D^{{\mars}}_{i,j}+p^{{\mars}}_i (1-p^{{\mars}}_j)][D^{{\venus}}_{i,j}+p^{{\venus}}_i p^{{\venus}}_j]$
	*AA*	$[D^{{\mars}}_{i,j}+p^{{\mars}}_i p^{{\mars}}_j][D^{{\venus}}_{i,j}+p^{{\venus}}_i p^{{\venus}}_j]$

Nine classes of two-locus genotypes (L1, L2) for offspring in a full-sib family and the probability of each class as a function of the parental LD *D* and the *A*-allele frequency $p_i$ at locus *i* (the superscripts indicate the sex of the parent and the subscripts indicate the locus)

The covariance between the additive genotype indicators in Eq. 3 is then determined by:

11 $$\begin{aligned} \text {cov}\left( c_{a,i},c_{a,j}\right)&= \sum _{s=1}^3 \sum _{q=1}^3 c_{a,s} c_{a,q} z_{s,q} \nonumber \\&\quad -\sum _{s=1}^3 c_{a,s} z_{s,_{\bullet}}\sum _{q=1}^3 c_{a,q} z_{_{\bullet},q}, \end{aligned}$$

where the dot indicates summation over all assigned components, and $z_{s,_{\bullet }}$ and $z_{_{\bullet },q}$ are the marginal genotype probabilities for the first and second locus, respectively. Furthermore, $c_{a,1}$ = −1 since the first row and the first column contain genotype *BB*; $c_{a,2}=0$ since the second row and the second column contain heterozygous genotypes; and by analogy, $c_{a,3}=1$. After simplification (using Mathematica [8]), the result obtained for the off-diagonal elements of the covariance matrix ${\varvec{\Omega}}$ is:

12 $$\text {cov}\left( c_{a,i},c_{a,j}\right) =D^{{\mars}}_{i,j}+D^{{\venus}}_{i,j}.$$

Note that the LD depends on the recombination fraction $\theta$, which can be converted into a genetic distance *x* (in Morgan) using Haldane’s mapping function [9]

13 $$x=-0.5 \ln (1-2\theta ).$$

To complete the covariance matrix ${\varvec{\Omega}}=\text {cov}(c_{i},c_{j})_{i,j}$, the variance in the genotype indicator (as defined by Eq. 3) at each locus *i* is expressed as a function of *A*-allele frequency $p_i$. The genotype frequencies at locus *i* are $(1-p_i^{{\mars}})(1-p_i^{{\venus}})$ for genotype *BB*, $p_i^{{\mars}}(1-p_i^{{\mars}})+p_i^{{\venus}}(1-p_i^{{\venus}})$ for *AB* / *BA*, and $p_i^{{\mars}} p_i^{{\venus}}$ for *AA*. Then,

14 $$\text {E}\left( c_{a,i}\right) =-1+p_i^{{\mars}}+p_i^{{\venus}}$$

and

15 $$\text {E}\left( c^2_{a,i}\right) =1-p_i^{{\mars}}-p_i^{{\venus}}+2p_i^{{\mars}} p_i^{{\venus}},$$

and thus

16 $$\text {var}\left( c_{a,i}\right) = \text {E}\left( c^2_{a,i}\right) -\text {E}^2\left( c_{a,i}\right) =\pi _i^{{\mars}} + \pi _i^{{\venus}}$$

with $\pi _i^{{\mars}}=p_i^{{\mars}}(1-p_i^{{\mars}})$ and $\pi _i^{{\venus}}=p_i^{{\venus}}(1-p_i^{{\venus}})$. Note that the only possible values for $\text {var}(c_{a,i})$ are 0, $\frac{1}{4}$, or $\frac{1}{2}$, since $\pi _i^{{\mars}}$ and $\pi _i^{{\venus}}$ can only have values of 0 or $\frac{1}{4}$.

Now we have derived all of the elements of the covariance matrix ${\varvec{\Omega}}$, so we can express the Mendelian variance for a particular trait as (Eq. 2):

$$\sigma ^2_g = {\mathbf{m}}^{\prime }{\varvec{\Omega }} {\mathbf{m}}.$$

As already mentioned, this Mendelian sampling variance can be split into the sum of two independent parental contributions:

17 $$\sigma ^2_g = {\mathbf{m}}^{\prime }{\varvec{\Omega}} {\mathbf{m}} = {\mathbf{m}}^{\prime } {\varvec{\Omega}}^{{\mars}} {\mathbf{m}} + {\mathbf{m}}^{\prime } {\varvec{\Omega}}^{{\venus}} {\mathbf{m}},$$

where $${\varvec{\Omega }}^{{\mars}}$$ and $${\varvec{\Omega }}^{{\venus}}$$ represent parent-specific covariance matrices for the additive effects of single alleles based on the paternal and maternal gametes. The paternal (maternal) covariance matrix $${{\varvec{\Omega }}^{{\mars}}}$$$$({{\varvec{\Omega }}^{{\venus}}})$$ contains off-diagonal elements that are equal to $$D_{i,j}^{{\mars}}$$ ($$D_{i,j}^{{\venus}}$$) and $$\pi _i^{{\mars}}$$ ($$\pi _i^{{\venus}}$$) on the diagonals, according to Eqs. 12 and 16.

The variance $\text {var}(c_{a,i})$ becomes zero at loci for which both parents are homozygous. The corresponding rows and columns of the covariance matrix only contain zeroes, which causes a rank deficiency. The corresponding diagonal and off-diagonal elements in the correlation matrix **R** are defined as zero (although they are not defined in a strictly mathematical sense) for our purposes in order to maintain rank equality between ${\varvec{\Omega }}$ and **R**.

### Joint additive and dominance genetic effects

The covariance between two dominance effects can be derived in a similar manner as that between additive effects. Again, we consider the genotype probabilities of Table 2 arranged in a three-by-three matrix ${\mathbf {Z}}=(z_{s,q})$ and we define: $c_{d,1}$ = −1 since the first row and the first column contain genotype *BB*; $c_{d,2}=1$ since the second row and the second column contain heterozygous genotypes; and by analogy, $c_{d,3}$ = −1. Inserting these dominance codes into Eq. 11 and expanding yield:

18 $$\begin{aligned} \text {cov}\left( c_{d,i},c_{d,j}\right)&=16D^{{\mars}}_{i,j}D^{{\venus}}_{i,j}\nonumber \\&\quad +4D^{{\mars}}_{i,j}\left( 1-2p^{{\venus}}_i\right) \left( 1-2p^{{\venus}}_j\right) \nonumber \\&\quad +4D^{{\venus}}_{i,j}\left( 1-2p^{{\mars}}_i\right) \left( 1-2p^{{\mars}}_j\right) . \end{aligned}$$

Analogous to the additive effects, for the variance of the dominance effects we obtain:

19 $$\text {var}\left( c_{d,i}\right) =4\left( \pi _i^{{\mars}} + \pi _i^{{\venus}}\right) -16\pi _i^{{\mars}} \pi _i^{{\venus}},$$

which can take values 1 or 0. In particular, $\text {var}(c_{d,i})$ is equal to 1 if at least one parent is heterozygous, and 0 if both parents are homozygous. Thus, we can conclude that if one marker is homozygous in both parents, the covariance between the dominance effects is equal to 0, which is analogous to previous considerations of the rank deficiency for additive effects. Furthermore, Eqs. 18 and 19 contain the products of the characteristic parameters ($D_{i,j},p_i,p_j,\pi _i$) for both the sire and the dam, which is why ${\varvec{\Omega }}$ and the Mendelian variance can no longer be split into a sum of two separate parental parts when dominance effects are included.

In order to determine the covariance $\text {cov}(c_{a,i},c_{d,j})$ between the additive and dominance indicators, we assign the additive indicators by Eq. 3 to the first (*i*th) locus and the dominance indicators by Eq. 4 to the second (*j*th) locus, i.e.,

20 $$\begin{aligned} \text {cov}\left( c_{a,i},c_{d,j}\right) & = \sum _{s=1}^3 \sum _{q=1}^3 c_{a,s} c_{d,q} z_{s,q}\nonumber \\ & \quad -\sum _{s=1}^3 c_{a,s} z_{s,_{\bullet }} \sum _{q=1}^3 c_{d,q} z_{_{\bullet },q},\end{aligned}$$

which has to be expanded, resulting in:

21 $$\text {cov}\left( c_{a,i},c_{d,j}\right) =2D^{{\mars}}_{i,j}\left( 1-2p^{{\venus}}_j\right) +2D^{{\venus}}_{i,j}\left( 1-2p^{{\mars}}_j\right) .$$

Exchanging the loci yields:

22 $$\begin{aligned} \text {cov}\left( c_{d,i},c_{a,j}\right)&=\text {cov}\left( c_{a,j},c_{d,i}\right) \nonumber \\&=2D^{{\mars}}_{i,j}\left( 1-2p^{{\venus}}_i\right) \nonumber \\&\quad +2D^{{\venus}}_{i,j}\left( 1-2p^{{\mars}}_i\right) . \end{aligned}$$

**Fig. 1:**
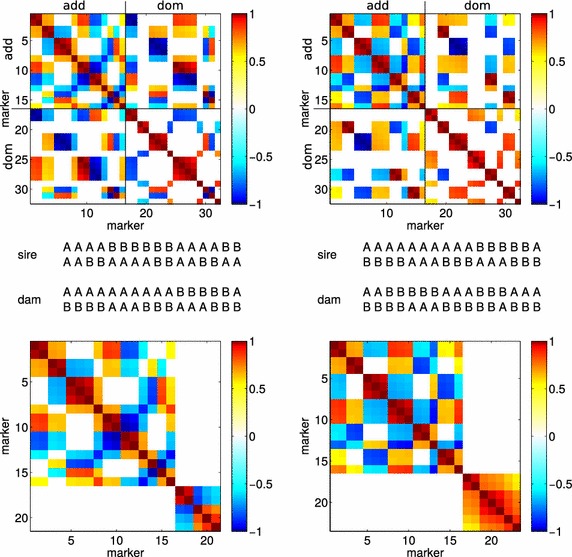
Correlation matrices for two matings (*top*) with their respective phased genotypes (*middle*). The marker distance is 1 cM. The dimension of both matrices is 32 due to the additive and dominance effects at the 16 markers. Block diagonal matrices (*bottom*) remain after the *rows* and *columns* of dominance effects are deleted when there is a perfect correlation between a dominance and additive effect at the same locus

The rank deficiencies in ${\varvec{\Omega }}$ arise from variances equal to 0 as well as from perfect correlations, which is demonstrated by the two examples of joint correlation matrices for additive and dominance effects shown in the upper part of Fig. 1, as well as their corresponding parental diplotypes. The number of markers is 16 in both examples, which yield correlation matrices with dimensions $32 \times 32.$ Some diagonal elements of the off-diagonal blocks, which contain correlations between additive and dominance effects, indicate a perfect dependency between the additive and dominance effects at the same locus with correlations of either 1 or −1. After the redundant rows and columns have been deleted from the dominance part of the matrix, the correlation matrices obtained (Fig. 1, bottom) exhibit a block diagonal structure. The remaining covariance matrix for dominance effects corresponds to loci for which both parents are heterozygous (five and seven for the example in Fig. 1). The first block remains unchanged numerically, but it has a different interpretation because it now represents the correlation matrix for a vector ${\mathbf{m}}_{a^*}$, which is defined as:

23 $$\begin{aligned} {\mathbf{m}}_{a^*}=\left[ \begin{array}{cc} {\mathbf {I}}_n&\quad {\mathbf{H}}_n \end{array} \right] \left[ \begin{array}{c} {\mathbf{m}}_a\\ {\mathbf{m}}_d \end{array} \right] , \end{aligned}$$

where ${\mathbf{m}}_a$ and ${\mathbf{m}}_d$ are vectors of the additive and dominance effects for all markers in order of their map position and ${\mathbf {I}}_n$ is an identity matrix of order *n*. ${\mathbf{H}}$ is a diagonal matrix with elements:

24 $$\begin{aligned} h_{i,i}=\left\{ \begin{array}{ll} 0, &{}\quad \text{if }\,\text{both} \,\text{parents } \,\text{are } \,\text{heterozygous } \\ &{}\quad \,\text{or } \,\text{homozygous }\\ 1, &{}\quad \text{if } \,\text{one } \,\text{parent } \,\text{is } \,\text{heterozygous } \\ &{}\quad \text{and } \,\text{the } \,\text{other } \,\text{is } BB \\ -1, &{}\quad \text{if } \,\text{one } \,\text{parent} \,\text{is } \,\text{heterozygous } \\ &{}\quad\text{and}\, \text{ the }\, \text{ other } \,\text{ is } AA \end{array}\right. . \end{aligned}$$

Now, $m_{a^*,i}$ has the form:

25 $$\begin{aligned} m_{a^*,i}=\left\{ \begin{array}{ll} m_{a,i}, &{}\quad \text{ if }\, \text{ both}\, \text{ parents}\, \text{ are } \\ &{}\quad \text{ heterozygous } \\ &{}\quad \text{ or } \,\text{ homozygous } \\ m_{a,i}+m_{d,i}, &{}\quad \text{ if }\, \text{ one }\, \text{ parent }\, \text{ is }\, \text{ heterozygous } \\ &{}\quad \text{ and }\, \text{ the }\, \text{ other }\, \text{ is } BB \\ m_{a,i}-m_{d,i}, &{}\quad \text{ if }\, \text{ one }\, \text{ parent }\, \text{ is }\, \text{ heterozygous }\\ &{}\quad \text{ and }\, \text{ the }\, \text{ other }\, \text{ is }\, AA \end{array} \right. . \end{aligned}$$

As a practical consequence, we can use ${\mathbf{m}}_d{^*}$ (i.e., ${\mathbf{m}}_d$ with all elements $m_{d,i}$ eliminated, where one parent is homozygous at locus *i*) and ${\mathbf{m}}_a{^*}$. Then, $\sigma ^2_{g}$ can be computed as:

26 $$\begin{aligned} \sigma ^2_{g}=\left[ \begin{array}{cc} {\mathbf{m}}_{a^*}^{\prime }&{\mathbf{m}}_{d^*}^{\prime } \end{array} \right] {\varvec{\Omega}}^*\left[ \begin{array}{c} {\mathbf{m}}_{a^*}\\ {\mathbf{m}}_{d^*}\end{array} \right] , \end{aligned}$$

where ${\varvec{\Omega }}^*$ has a block-diagonal structure with unequally sized blocks, as in the example shown in Fig. 1.

### Mendelian covariance with multiple traits

The Mendelian covariance between traits $(t_k,t_l)$ is also of interest, especially when we aim at determining the Mendelian variance of the aggregate genotype (multiple trait breeding goal). If ${\mathbf{m}}_k$ and ${\mathbf{m}}_l$ are the vectors of marker effects for traits *k* and *l*, then the Mendelian covariance $\sigma _g(t_k,t_l)$ between these two traits is

27 $$\sigma _g\left( t_k,t_l\right) ={\mathbf{m}}_k^{\prime }{\varvec{\Omega }} {\mathbf{m}}_{l}.$$

The Mendelian variances and covariances for several traits can be collected in the Mendelian covariance matrix:

28 $$\begin{aligned} \mathbf V = \left[ \begin{array}{cccc} \sigma ^2_g(t_1)&{}\quad \sigma _g(t_1,t_2)&{}\quad \cdots &{}\quad \sigma _g(t_1,t_N)\\ \sigma _g(t_1,t_2)&{}\quad \sigma ^2_g(t_2)&{}\quad \cdots &{}\quad \sigma _g(t_2,t_N)\\ \vdots &{}\quad \vdots &{}\quad \ddots &{}\quad \vdots \\ \sigma _g(t_1,t_N)&{}\quad \sigma _g(t_2,t_N) &{}\quad \cdots &{}\quad \sigma ^2_g(t_N)\\ \end{array}\right] , \end{aligned}$$

which is given by:

29 $${\mathbf {V}}={\mathbf {M}}^{\prime } {\varvec{\Omega }} {\mathbf {M}},$$

where each column ${\mathbf{m}}_k$ of **M** is a vector of marker effects for trait *k*. The Mendelian sampling variance for the aggregate genotype can then be obtained from **V** and the vector **f** of the economic weights for all traits:

30 $$\sigma ^2_{gT}={\mathbf {f}}^{\prime } {\mathbf {V}} {\mathbf {f}}.$$

This quantity has a pivotal role in mating decisions because the total breeding value (defined as the linear combination of single breeding values ${\mathbf {f}}^{\prime }{\mathbf {t}}$, ${\mathbf {t}}=(t_1,\ldots ,t_N)$) is the most important criterion for selection.

## Practical application

We compared the exact method with a recently published simulation approach [5]. The Mendelian sampling variance of gametes was calculated with both methods for each animal from a dataset that included the diplotypes of 74,353 male and female German Holstein cattle. Identical sets of recombination rates and estimates of additive marker effects were used for both methods. These parameters were derived from routine genomic evaluation data. This comparison was done for four traits: fat yield (FKG), protein yield (PKG), somatic cell score (SCS), and the direct genetic effect on stillbirth (SBd).

The exact gametic Mendelian variances $\sigma ^2_{\hat{g}}$ of the estimated gametic values were calculated for each trait and each animal as:

31 $$\sigma ^2_{\hat{g}}= {\hat{\mathbf{m}}}^{\prime } {{\varvec{\Omega}}^{{\mars}}} {\hat{\mathbf{m}}}\quad \text {or} \quad \sigma ^2_{\hat{g}}= {\hat{\mathbf{m}}}^{\prime } {{\varvec{\Omega}}^{{\venus}}} {\hat{\mathbf{m}}}$$

according to Eq. 2, where ${\hat{\mathbf{m}}}$ is the vector of the estimated additive marker effects. The simulation method used 100,000 randomly generated gametes for each animal. Thus, the estimate of the Mendelian variability for the estimated gametic values was:

32 $${\widehat{\sigma ^2}}_{\hat{g}}=\sum _{o=1}^{K}\frac{\left( {\mathbf {w}}_o^{\prime } {\hat{\mathbf{m}}}\right) ^2}{K} -\left( \sum _{o=1}^{K} \frac{{\mathbf{w}}_o^{\prime }{\hat{\mathbf{m}}}}{K}\right) ^2,$$

where *K* = 100,000 is the number of simulated gametes per individual and ${\mathbf {w}}_o$ is the vector of the genotype indicators for the *o*th simulated gamete in the individual under consideration.

The Mendelian covariances between traits were also obtained using the exact method by applying Eq. 29. Furthermore, the four traits were combined with weights of 0.1, 0.4, 0.375, and 0.175 for the traits FKG, PKG, SCS, and SBd, respectively, and the (gametic) Mendelian variances were computed for this aggregate genotype. The covariances and aggregate genotypes were not implemented in the simulation method, so no comparisons could be made with the simulation method for these quantities.

## Results

**Fig. 2:**
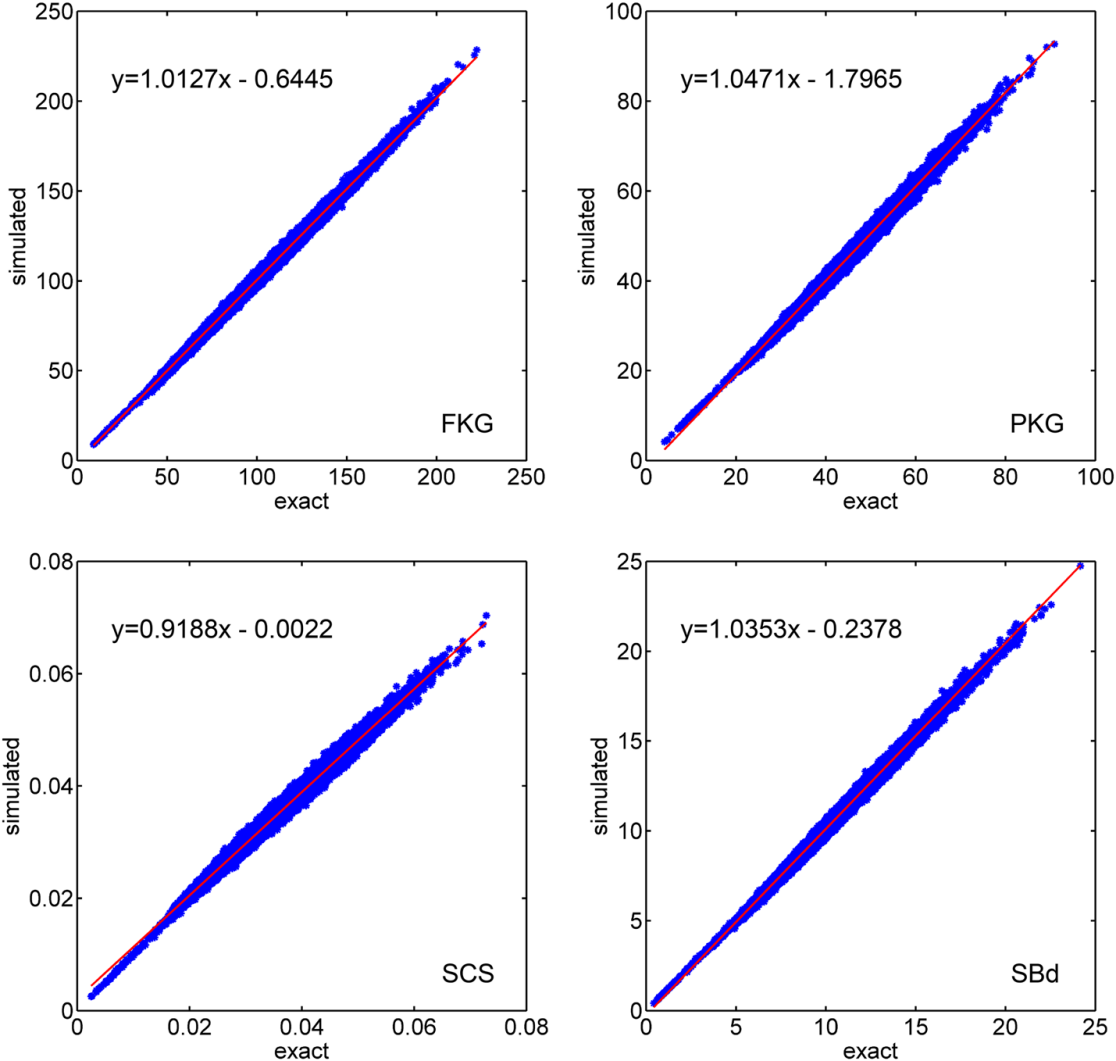
*Scatter plots* of Mendelian gametic variances obtained from simulation versus the exact values. Differences between simulated values (each one derived from 100,000 simulated gametes per candidate) and exact values appear as vertical deviations from the linear regression lines (in *red*) and represent Monte Carlo errors. The traits include fat yield (FKG), protein yield (PKG), somatic cell score (SCS), and direct genetic effect on stillbirth (SBd)

Scatter plots of the estimated ${\widehat{\sigma ^2}}_{\hat{g}}$-values against their exact $\sigma ^2_{\hat{g}}$ counterparts are in Fig. 2. The slopes of the linear regression lines were close to unity for all of the traits and the intercepts were small, which indicated a good average agreement between both methods. The variation around the regression line was due to the Monte Carlo error of the simulation.

**Fig. 3:**
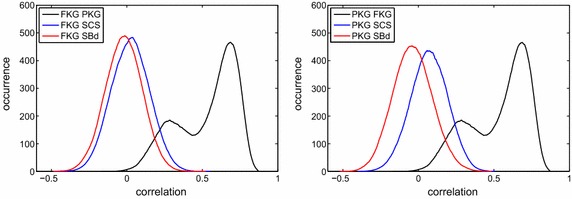
Frequency distributions of the Mendelian correlations. The frequency distributions of the correlations between FKG and all the other traits (PKG, SCS, and SBd) are shown on the *left*, and those between PKG and all the other traits are shown on the *right*. *FKG* fat yield, *PKG* protein yield, *SCS* somatic cell score, and *SBd* direct genetic effect on stillbirth

To facilitate a better comparison between the trait combinations, the Mendelian covariances were transformed into correlations and their distributions are in Fig. 3. Interestingly, the sign and magnitude of these correlations exhibited a very high amount of variation between individuals. The Mendelian correlation between FKG and PKG was an exception because most of the values were positive and the distribution was bimodal. This bimodality is a consequence of the *DGAT1* gene, for which heterozygous animals led to the smaller peak at correlations below 0.5 and homozygotes were responsible for the larger peak with correlations above 0.5.

The coefficient of variation of the Mendelian variances of the aggregate genotype was 18.7 %, which was similar to the coefficient of variation of the Mendelian variances of PKG (19.0 %), SCS (20.2 %), and SBd (21.5 %), but somewhat smaller than that of FKG (30.3 %).

## Discussion

**Fig. 4:**
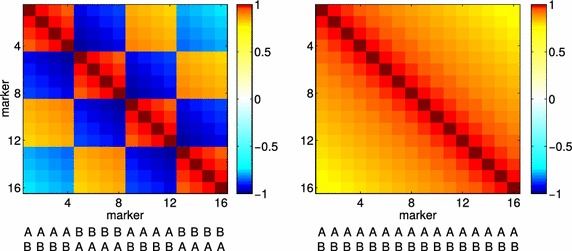
Parent-specific correlation matrix ${\varvec{\Omega}}^{{\mars}}$ of the gametes. The respective phased genotypes are listed below. All markers are heterozygous, with a genetic distance of 1 cM between markers. For the first animal (*left*), markers 1–4 and 9–12 are in coupling phase, whereas markers 5–8 and 13–16 are in repulsion phase with respect to the first marker. For the second animal (*right*), all markers are in coupling phase

The derived covariance matrices depend on prior information on the order and genetic distance of markers, as well as the parental diplotypes. They allow the calculation of the within-family variation of the estimated genetic values based on exact considerations of the genotypes, degrees of homozygosity, and linkage phases in the parents. In the classical formula, $\frac{1}{4}\sigma ^2_a (1-F_{{\mars}})+ \frac{1}{4}\sigma ^2_a (1-F_{{\venus}})$, the loss of homozygosity in the parents is considered relative to the base population, where inbreeding coefficients are zero and the Mendelian variance is at maximum ($\frac{1}{2}\sigma ^2_a$) for all families. Our covariance matrices, in contrast, mirror the absolute level of marker homozygosity and the Mendelian variance reaches its maximum for fully heterozygous parents with all positive marker alleles in coupling phase. Different linkage phases can make a substantial difference in marker covariability, as shown by the example in Fig. 4, which presents the correlation matrices for two diplotypes with identical 16-marker genotypes but different linkage phases.

### Fields of application and computational aspects

In general, the method described in this study can be applied to all diploid animals and plants. Of course, all relevant input parameters must be known, such as the marker maps, marker effects, and phased genotypes. Crosses of double haploid (i.e. fully inbred) lines occur as parents in breeding programs for plant species such as e.g. maize. An advantage of such parents is that they provide reliable diplotypes because of the complete homozygosity of genotyped grandparents, whereas the derivation of diplotypes is prone to some degree of phasing error in non-inbred populations [10]. In cases where the phase of some SNPs is only known at a probabilistic level, it may be an option to average the Mendelian sampling variances over all possible linkage phases. Simplifications may be possible, e.g. by taking only the most probable diplotypes into account. However, we did not investigate this question in detail.

For humans and mice, it has been found that marker maps generally differ for male and female parents [11]. All the covariances can be adjusted easily for sex-specific recombination rates, which is achieved most easily in the pure additive case by applying the male and female recombination rates to set up ${\varvec{\Omega }}^{{\mars}}$ and ${\varvec{\Omega }}^{{\venus}}$. In the general case, the LD measures for both the paternal and maternal gametes $D^{{\mars}}$ and $D^{{\venus}}$ must be adjusted in order to obtain the adjusted covariances.

The sex of the full sibs matters for the inclusion of sex chromosomes because when all considered progeny are female, the sire can be treated as homozygous at all X-chromosomal loci and the calculation can proceed as usual. When the focus is on male progeny, such as young bulls obtained from elite matings, there is no X-chromosomal paternal contribution to the Mendelian sampling variance. Of course, dominance has no effect on the X-chromosomal Mendelian variance in males, unlike for females.

From a computational viewpoint, the purely additive case is most convenient because the parental contributions to the Mendelian variances and covariances can be calculated for a large list of potential parents and all traits by setting up the parent-specific covariance matrix. Subsequently, the parental contributions only have to be added to the total within-family variance for each mating considered. The computational time required by the exact method was roughly the same as that for the simulation approach. However, the computational demand would increase for the latter case if the Monte Carlo error needs to be reduced further.

Population-averaged Mendelian sampling variances for single traits were previously derived from large numbers of phased genotypes and available estimates of additive marker effects by Cole and VanRaden [6]. Neither simulation of gametes nor covariance matrices were used in this study since loci on the same chromosome were either assumed to be perfectly linked or fully independent. Consequently, their respective results can only be interpreted as upper and lower limits. In another study, Segelke et al. [5] took recombination within chromosomes into account by simulating gametes of individuals with known diplotypes. Parental contributions to the within-family additive genetic variance were expressed as standard deviations of gamete breeding values in a family-specific manner.

Consideration of the aggregate genotype calls for a full Mendelian sampling covariance matrix across traits, which, in the additive case, can also be derived by simulation, but this has not yet been reported in the literature. This requires that genomic breeding values are estimated for each trait of interest and each single simulated gamete and then averages of squares and cross-products are calculated over gametes. If dominance effects are to be included, pairs of paternal and maternal gametes have to be simulated. The simulation-inherent Monte Carlo errors of all single-trait variances and all pair-wise covariances will, of course, propagate and induce a joint Monte Carlo error of the resulting variability in the aggregate genotype.

From a producer’s perspective, phenotypic uniformity of a population of plants or animals is desirable because it facilitates management. Matings with high additive genetic merit and low within-family genetic variance [6] may be attractive to achieve that goal. Dominance—if of some importance for the traits under consideration—could be included for the same purpose. Breeding organizations, in contrast, are probably more interested in offspring with exceptionally high breeding values [6], since e.g. in dairy cattle, semen prices are non-linearly related to the genetic merit of bulls. For a particular mating, the probability that the estimated breeding value of offspring will be greater than a certain threshold can be determined from a normal distribution with family-specific mean and variance. The opportunity to breed the desired animals of top genetic merit can then be maximized by choosing the matings with the highest probabilities among all possible matings, possibly by taking some constraints such as inbreeding into account.

The average observed degree of homozygosity in the German Holstein dataset was 65.3 %, with a range from 25.2 to 88.0 %. These high degrees of homozygosity were exploited for computational speed by deleting the rows and columns for homozygous markers from the covariance matrix and the respective marker effects from ${\hat{\mathbf{m}}}$. Therefore, the dimensions of the remaining vector of marker effects and the remaining covariance matrix were reduced greatly, leading to considerable computational time savings. Note that both parents had to be homozygous in the dominance case in order to reduce matrix dimension in a similar way. Clearly, computational time can be decreased by implementing parallel calculation of individuals and chromosomes. However, in the presence of dominance, each considered mating must be computed with its own covariance matrix, and thus only matings can be parallelized (the chromosomes are unaffected).

### Choosing alternative genotype indicators

The formulae for the covariances and correlations are functions of the chosen genotype indicators, for which different options exist [12]. In the present study, we used (1, 0, −1) (Eq. 3) for additive effects and (−1, 1,−1) (Eq. 4) for dominance effects, but other possible indicators include (0, 1, 2) for additive effects and (0, 1, 0) or $\left( -\frac{1}{2},\frac{1}{2},-\frac{1}{2}\right)$ for dominance effects. All these indicators can be transformed into each other by a shift and/or a multiplication by a constant. Simply shifting the indicators does not influence the (co)variance, so it is also possible to use the formulae described in this study when additive marker effects have been estimated via the (0, 1, 2) coding.

However, multiplication of genotype codes by a constant factor does affect the (co)variance. For example, let ${\mathbf{m}}_{\tilde{d}}$ be the dominance marker effect when the indicator $c_{{\tilde{d}},i}= (0,1,0)$ is used for its estimation. The Mendelian sampling variance is then calculated by ${\mathbf{m}}^{\prime }_{a,{\tilde{d}}}{\varvec{\Omega }}^{}_{a,{\tilde{d}}}{\mathbf{m}}^{}_{a,{\tilde{d}}},$ where ${\mathbf{m}}^{\prime }_{a,\tilde{d}}$ is known, but ${\varvec{\Omega }}^{}_{a,\tilde{d}}$ is not known. The type of parameterization does not affect the Mendelian sampling variance, so:

33 $${\mathbf{m}}^{\prime }_{a,{\tilde{d}}}{\varvec{\Omega }}^{}_{a,{\tilde{d}}}{\mathbf{m}}^{}_{a,\tilde{d}}={\mathbf{m}}^{\prime }_{a,d}{\varvec{\Omega }}^{}_{a,d}{\mathbf{m}}^{}_{a,d}$$

must hold, where the terms on the right-hand side are known. The relationship between indicators $c_{\tilde{d}}$ and $c_{d}$ is $c_{\tilde{d},i}=\frac{c_{d,i}+1}{2}$; hence,

34 $$\begin{aligned} \text {cov}\left( c_{{\tilde{d}},i},c_{{\tilde{d}},j}\right)&=\text {cov}\left( \frac{c_{d,i}+1}{2},\frac{c_{d,j}+1}{2}\right) \nonumber \\&=\frac{1}{4}\text {cov}\left( c_{d,i},c_{d,j}\right) , \end{aligned}$$

35 $$\begin{aligned} \text {cov}\left( c_{\tilde{d},i},c^{}_{a,j}\right)&=\text {cov}\left( \frac{c_{d,i}+1}{2},c_{a,j}\right) \nonumber \\&=\frac{1}{2}\text {cov}\left( c_{d,i},c_{a,j}\right) , \end{aligned}$$

and

36 $$\begin{aligned} \text {cov}\left( c^{ }_{a,i},c_{\tilde{d},j}\right)&=\text {cov}\left( c_{a,i},\frac{c_{d,j}+1}{2}\right) \nonumber \\&=\frac{1}{2}\text {cov}\left( c_{a,i},c_{d,j}\right) , \end{aligned}$$

and thus the Hadamard product

37 $${\varvec{\Omega }}_{a,\tilde{d}}={\mathbf {U}} \odot {\varvec{\Omega }}_{a,d}$$

with

38 $$\begin{aligned} \mathbf {U}=\left[ \begin{array}{cc}{\mathbf {1}}&{}\quad \frac{1}{2}{\mathbf {1}}\\ \frac{1}{2}{\mathbf {1}} &{}\quad \frac{1}{4}{\mathbf {1}}\end{array}\right] \end{aligned}$$

transforms the ${\varvec{\Omega }}_{a,d}$ matrix into ${\varvec{\Omega }}_{a,\tilde{d}}$. Instead of transforming the covariance matrix ${\varvec{\Omega }}$, the estimated marker effects can also be transformed, which can be implemented easily by multiplying the marker effects ${\mathbf{m}}_{\tilde{d}}$ by $\frac{1}{2}$.

Genetic differences can be parameterized in terms of substitution effects and dominance deviations, or in terms of additive and dominance genotype effects, as discussed in detail by Vitezica et al. [12]. For the former, the ${\hat{\mathbf{m}}}$-vector comprises estimates of the allele substitution effects ${\mathbf{m}}_{\alpha }$ and dominance effects ${\mathbf{m}}_{\tilde{d}}$. Allele substitutions effects are defined as (e.g. [13]):

39 $$m_{\alpha ,i}=m_{a,i}+m_{\tilde{d},i}(v_i-u_i),$$

where $u_i$ and $v_i=1-u_i$ are the population frequencies for alleles *A* and *B*, respectively. When only allele substitution effects ${\mathbf{m}}_\alpha$ are considered and dominance effects are ignored (e.g. [5]), it is possible to use the covariance matrix described in this study without changes.

If dominance effects are not ignored, the allele substitution effects must be transformed into additive marker effects in order to allow the use of the derived covariance matrix. This transformation is achieved by:

40 $$\begin{aligned} m_{a,i}&=m_{\alpha ,i}-m_{\tilde{d},i}(v_i-u_i)\nonumber \\&=m_{\alpha ,i}-\frac{1}{2}m_{d,i}(v_i-u_i). \end{aligned}$$

The allele frequencies *u* and *v*, which are required for the transformation, are already available because they are required for routine estimation.

## Conclusions

In this study, we proposed a new method for the exact calculation of Mendelian sampling (co-)variances based on knowledge of phased marker genotypes and marker effect estimates and we derived all the requisite formulae. The method considers inbreeding but also the absolute level of homozygosity, as indicated by the marker genotypes, while it also considers the linkage phase of the markers in both parents.

We demonstrated the applicability of our method by comparing its results with results produced by an established simulation method using a large dairy cattle dataset. We found that both approaches agreed within the range of the Monte Carlo error, which is inherent in the simulation, but which can be fully avoided because the derived covariance matrices represent an infinitely large number of progeny.
